# Revaluating the dietary methionine requirements in an improved strain of nile tilapia (*Oreochromis niloticus*)

**DOI:** 10.1016/j.heliyon.2023.e14376

**Published:** 2023-03-07

**Authors:** Rodrigue Yossa, Nurulhuda Ahmad Fatan, Kirthigah Palanivelu

**Affiliations:** Sustainable Aquaculture, WorldFish, Jalan Batu Maung, Batu Maung, 11960 Bayan Lepas, Penang, Malaysia

**Keywords:** Amino acids, Feed, Fish, Improved strain, Nutrient requirements, Nutrition, Tilapia

## Abstract

It has been shown with terrestrial animals that the genetic improvement increases the nutrient requirements of the animal, which becomes more efficient in using these nutrients to achieve their higher growth potential. This study was conducted to estimate the dietary methionine requirements of different generations of the Genetically Improved Farmed Tilapia (GIFT) strain of Nile tilapia at the juvenile stage (17.51 g–19.55 g initial body weight). To achieve this objective, a completely randomized 2 × 6 factorial design was applied, with the genetic background of fish (generation) as the first independent variable with two levels, the 16th and 17th generations, and the methionine content of the diet as the second independent variable, with 6 graded levels, 0.52, 0.62, 0.84, 0.94, 1.04 and 1.27% of methionine in the diets, respectively. At the end of the 42-day experiment, the interaction effect of the generation × diet was not significant (P > 0.05) for any of the response parameters studied. The genetic improvement led to 15% more growth in the 17th generation than the 16th generation of the GIFT strain of Nile tilapia, which was accompanied with better feed conversion ratio, protein productive value, and energy productive value in the former generation (P < 0.05). Whatever the generation considered, the optimum dietary methionine requirement for the growth of the improved GIFT strain of Nile tilapia was estimated in this study, using the broken-line model, between 0.75% and 0.80% of the diet (with a cysteine level of 0.50% of the diet), which is higher than 0.49% of the diet previously estimated for conventional Nile tilapia at the juvenile stage. Therefore, the genetic improvement applied to the GIFT strain of Nile tilapia likely led to a higher methionine and total sulfur amino acid requirement than the conventional strains. The results of this study support the need to update the existing nutrient requirement databases of fish, in order to ensure that diets are formulated to effectively satisfy the requirements of not only conventional, but also improved strains of fish, for the full expression of their accrued growth potential of the latter strains.

## 1 Introduction

Methionine is essential in fish and is together with lysine among the main limiting amino acids in either unbalanced diets or diets that contain high levels of plant-based protein sources [14]. To avoid signs of methionine deficiency such as reduced growth and feed efficiency, commercial forms (crystalline or intact) of methionine are successfully used in fish feeds to satisfy the known requirement levels in fish. This level was estimated at 0.8% of the diet (at the cystine level of 0.2% of the diet) for Nile tilapia (*Oreochromis niloticus*) fry of 0.06 g initial body weight by Ref. [18] in 1988, which coincidentally is exactly the year selective breeding project for the Genetically Improved Farmed Tilapia (GIFT) strain of Nile tilapia started in the Philippines [3,15]. This methionine requirement level was later estimated at 0.49% of the diet (at cysteine level of 0.45% of the diet) for juvenile Nile tilapia of 1.3 g initial body weight by Ref. [13]. This figure is still recommended today for juvenile Nile tilapia and many other strains and species of tilapias farmed globally [14]. However, it has been showed that the genetic improvement increases the nutrient requirements of an animal, which becomes more efficient in using these nutrients to achieve their higher growth potential [4]. Nevertheless, only a few studies have been conducted focusing specifically on re-evaluating nutrient requirements of improved strains of farmed fish. It has been observed in Nile tilapia that the fatty acid requirements of the improved strains are higher than those of the conventional strains [12]. Such information does not exist on the amino acid requirements, and it would be informative to assess whether the genetic improvement shifts the methionine requirement level to support the higher growth. This information would be used to update the existing nutrient requirement database, in order to ensure a better feed formulation targeting the improved strains for farmed fish. The objective of this study was therefore to estimate the dietary methionine requirements of different generations of the GIFT strain of Nile tilapia at the juvenile stage.

## 2 Materials and methods

### 2.1 Experimental context

The experiment was conducted at the Aquaculture research facility of WorldFish, Penang, Malaysia. The research facility comprised thirty-six 40 L aquaria mounted in a recirculating aquaculture system (RAS) with water temperature maintained around 28 °C and a water flowrate set at 2 L/min in each aquarium (i.e. 3 water exchanges per hour per aquarium) throughout the duration of the experiment. Individual air-stones connected to a blower delivered air in each aquarium to maintain a dissolved oxygen (DO) level between 4.62 and 5.8 mg/L. The pH was maintained between 6.4 and 7.1. The water temperature, DO and pH were recorded in 10 random aquaria and the sump daily, at 11.30 (about 3 h following the morning feeding). The nitrite (NO^−2^), nitrate (NO^−3)^ and NH^3^ contents in the water were measured in the sump and random tank three times a week and maintained at 0.01–0.3 mg/L, 0.8–1.6 mg/L and 0.4–0.9 mg/L, respectively, throughout the experiment. The RAS was housed within a room illuminated with fluorescent lights, which were automatically set for continuous 12-h light per day.

### 2.2 Experimental design and facility

The experiment was conducted following a completely randomized 2 × 6 factorial design. The genetic background of fish (generation) was the first independent variable with two levels, the 16th and 17th generations of the improved GIFT strain of Nile tilapia. The second independent variable was the methionine content of the diets, with 6 graded levels, namely, 0, 0.1, 0.3, 0.4, 0.6, and 0.8% of methionine in 6 experimental diets (at the expense of glutamic acid). The analysed values of the methionine contents of these diets were 0.52, 0.62, 0.84, 0.94, 1.04 and 1.27% respectively. These diets were referred to as M0.52, M0.62, M0.84, M0.94, M1.04 and M1.27, respectively, throughout this article. The 2 × 6 factorial combination led to 12 treatments. Each treatment was applied in triplicate, for a total of 36 experimental units (aquaria). The experiment lasted 42 days.

### 2.3 Fish

The fish were handled in accordance with the guidelines of the Research Animal Ethics Committee of the National University of Malaysia (UKMAEC), which provided the approval number WORLDFISH/2020/RODRIGUE YOSSA/14-MAY/1108-MAY-2020-SEPT.-2020. Two batches of 1000 mix-sex fingerlings of the GIFT strain of Nile tilapia of each of the two experimental generations (16th and 17th) was obtained from the Genetic Group of WorldFish. The fish were acclimated in the research facility for two weeks prior to the start of the experiment. During the acclimation period, an equal number of fish were distributed to the aquaria and fed a commercial tilapia feed (Cargill pre starter 6103) during the first week and the M0.52 diet during the second week. The day before the start of the experiment, fish were not fed and pooled to a 1-m^3^ tank before they been randomly distributed to the experimental tanks. Ten fish were stocked in each of the 40-L aquarium. The average initial body weight was 17.51 g and 19.55 g for the 16th and 17th generations, respectively. The experiment effectively started at the stocking.

### 2.4 Experimental diets (treatments)

Six experimental diets (Table 1) were formulated to meet the known nutrients requirements of Nile tilapia [14], except for the methionine that were below the recommended level for the diets M0.52, M0.62. The diet M0.84 was formulated to provide exactly the required dietary methionine level of Nile tilapia [18], whereas the diets M0.94, M1.04 and M1.27 were formulated to provide more dietary methionine than the requirement level. The diet preparation followed the process that was thoroughly described in Ref. [21]. Briefly, the dry ingredients of each of the experimental diets were milled to <750μm particle size, then oil was added and the mash was mixed in a food mixer (Model B–20 N, THE BAKER; Malaysia) for 20 min. The hot water (35–45% of the diet mix) was then added to the mash to make a dough, which was transferred to a Professional Pasta Machine (Model Dolly, La Monferrina; Italy) to produce feed pellets (diameter = 1.9 mm, length = 2.5 mm; die number 9). The pellets were dried at 60 °C using a VENTICELL dryer (Model LSIS-B2V/VC 404, MMM Medcenter Einrichtungen GmbH; Germany) for 6 h. The experimental diets were packaged in labelled zipper bags and stored in the freezer at −20 °C until use. The chemical composition of each experimental diet and their amino-acid content were analysed and presented in Table 1, Table 2, respectively.

**Table 1:** Macronutrient composition of the six experimental diets containing graded levels of dietary methionine, fed for 42 days to two generations of improved juvenile Nile tilapia, with average initial body weights of 17.51 g and 19.55 g for the 16th and 17th generations, respectively.

	Experimental diets^a^					
Ingredients^b^ (%)	M0.52	M0.62	M0.84	M0.94	M1.04	M1.27
Distillers dried grains with solubles, DDGS	19.75	19.75	19.75	19.75	19.75	19.75
Corn gluten meal, 50% CP	17	17	17	17	17	17
Soybean meal, 48% CP, solvent extracted	25.1	25.1	25.1	25.1	25.1	25.1
Corn meal	19.7	19.7	19.7	19.7	19.7	19.7
Wheat bran	6.4	6.4	6.4	6.4	6.4	6.4
Canola oil	2	2	2	2	2	2
Fish oil	3	3	3	3	3	3
Dicalcium phosphate, DCP, CaHPO4	3	3	3	3	3	3
Mineral premix	1	1	1	1	1	1
Rovimix-stay-C 25, ascorbyl-monophosphate	0.15	0.15	0.15	0.15	0.15	0.15
Vitamin premix	0.75	0.75	0.75	0.75	0.75	0.75
Soy lecithin	0.5	0.5	0.5	0.5	0.5	0.5
L-Threonine	0.25	0.25	0.25	0.25	0.25	0.25
L-Lysine	0.6	0.6	0.6	0.6	0.6	0.6
L-glutamic acid	0.8	0.7	0.5	0.4	0.2	0
DL-Methionine	0	0.1	0.3	0.4	0.6	0.8
***Total***	100	100	100	100	100	100
***Analysed composition***						
**Dry matter, dm (g/100g)**	96.4	96.2	95.5	96.1	95.7	94.6
**Crude protein (g/100 g dm)**	35.58	35.55	36.02	35.9	36.26	36.58
**Crude lipid (g/100 g dm)**	10.89	10.91	10.89	10.93	10.27	10.68
**Ash (g/100 g dm)**	7.54	7.47	7.62	7.69	7.77	7.88
**Crude Fibre (g/100 g dm)**	4.5	4.32	4.43	4.41	4.32	4.48
**Carbohydrate (g/100 g dm)**^c^	45.99	46.06	45.47	45.48	45.69	44.87
**GE (kcal/100 g dm)**	424.27	425.16	424.08	423.52	420.06	421.78

a M0.52, M0.62, M0.84, M0.94, M1.04 and M1.27 refer to the 6 experimental diets in which 0, 0.1, 0.3, 0.4, 0.6 and 0.8% of methionine were added at the expense of the glutamic acid, leading to analysed methionine levels of 0.52, 0.62, 0.84, 0.94, 1.04 and 1.27% of methionine in the diets, respectively.

b The wheat bran, fish oil, dicalcium phosphate, mineral mix, vitamin mix, soy lecithin, soya bean meal, corn meal, canola oil, rovimix-stay-C 25 ascorbyl-monophosphate and DL-methionine were obtained from Sri Purta Trading Sdn Bhd 103, Kawasan Perusahaan Tandop Baru, Jalan Susuran Tandop, 05400, Alor Star, kedah, Malaysia; the corn gluten was obtained from PK-Agro industrial Products (M) Sdn Bhd, Lingkaran Sultan Hishamuddin, KWS 20, Selat Klang Utara, 42,000 Port Klang, Malaysia; the L-threonine and L-lysine was obtained from Yenher Agro-products Sdn Bhd, 1628 Jalan IKS Simpang Ampat 1, Taman IKS Simpang Ampat, 14,100 Simpang Ampat, S.P.S., Malaysia; the L-glutamic acid was obtained from Sigma-Aldrich (M) Sdn, Bhd, Level 3, Menara Sunway Annexe, Jalan Lagoon Timur, Bandar Sunway, 46,150 Petaling Jaya, Malaysia; the distillers dried grains with solubles (DDGS) was provided by U.S. Grains Council, 14-1 Wisma UOA Damansara II | No. 6 Changkat Semantan, Damansara Heights 50,490 Kuala Lumpur, Malaysia.

c Total carbohydrate content calculated as: total carbohydrate (% dry matter) = 100 – ash (%dm) – crude protein (% dm) – lipid (% dm).

**Table 2:** Amino acid composition of the six experimental diets containing graded levels of dietary methionine, fed for 42 days to two generations of improved juvenile Nile tilapia, with average initial body weights of 17.51 g and 19.55 g for the 16th and 17th generations, respectively.

Experimental diets^a^						
Amino acid (%w/w dm)	M0.52	M0.62	M0.84	M0.94	M1.04	M1.27
Essential Amino Acids						
Arginine	1.14	1.25	1.36	1.35	1.36	1.27
Histidine	1.45	1.46	1.88	1.77	1.78	1.8
Isoleucine	0.31	0.31	0.42	0.42	0.42	0.42
Leucine	3.53	3.53	4.29	3.95	3.97	3.91
Lysine	1.24	1.35	1.33	1.32	1.34	1.25
Methionine	0.52	0.62	0.84	0.94	1.04	1.27
Phenylalanine	1.87	1.98	2.62	2.5	2.93	2.85
Threonine	3.53	3.64	3.04	1.98	1.78	2.01
Valine	1.24	1.25	1.78	1.66	1.67	1.69
Tryptophan	0	0	0	0	0	0
Hydroxyproline	0	0	0	0	0	0
Glutamine	0	0	0	0	0	0
**Non-Essential Amino Acids**						
Alanine	2.28	2.29	2.62	2.91	3.03	2.85
Asparagine	0	0	0	0	0	0
Aspartic Acid	N.D.^b^	3.01	0.73	1.35	1.46	1.69
Glycine	1.24	1.25	1.36	1.14	1.25	1.06
Glutamic Acid	7.71	6.65	6.54	6.56	6.48	5.92
Cysteine	0.51	0.52	0.5	0.5	0.52	0.5
Proline	4.46	4.78	4.82	4.58	4.49	4.55
Serine	1.56	1.56	1.88	1.66	1.67	1.59
Tyrosine	1.56	1.46	1.68	1.46	1.78	1.37

a For information on the diets, please see the footnote of Table 1.

b N.D.: Non detectable, <0.01.

### 2.5 Feeding

The fish were hand-fed to apparent satiety three times daily (7–8am; 12-1pm; 4–5 pm), as determined by the loss of feeding activity after being offered food on at least three independent feeding episodes. During each feeding episode, the distribution of feed was done within 1 h. The amount of feed fed to each aquarium was recorded daily. Following each feeding, all uneaten feed was collected from each aquarium by siphoning, put in the labelled plastic bag, and kept frozen at −20 °C until the end of the experiment, when they were dried and weighed to allow for the accurate calculation of apparent feed intake.

### 2.6 Sample collection and analytical methods

Sampling occurred at the start, during the course and at the end of the experiment. At the start of the experiment, 40 fish of each genetic background were weighed and measured to the nearest millimetre from the pooled population to obtain a population size estimate. Two pools of 30 fish each were collected from each genetic background (4 samples) euthanized using an overdose of clove oil (400–500 mg/L). These fish pools were frozen at −20 °C and later analysed to obtain data on the proximate composition (DM, crude protein, crude lipid, ash, crude fiber and gross energy) of the base fish populations. The body, liver and gonad weights of 20 other fish were recorded for the calculation of the hepato-somatic and gonadosomatic indexes of the base fish populations. Prior to introducing the experimental fish in each aquarium, they were individually weighed and their total lengths recorded for the calculation of the initial condition factors in each tank.

During the experiment, fish mortalities were recorded and the weight of each dead animal recorded. Intermittent samplings were done at weeks 2, 4, and 6, and weights and counts were recorded in each tank. During the initial and intermittent samplings, the experimental fish were anaesthetized using clove oil (40–50 mg/L) prior to weighing, and then allowed to recover in their allocated tank following weighing. The fish were not fed during the sampling day.

At the end of the experiment, the fish from each tank were killed with an overdose of clove oil (400–500 mg/L), individually weighed, and their total lengths recorded for the calculation of the final weights and condition factors. Five fish from each aquarium were dissected and their liver and gonad weighed for the calculation of their individual hepato- and gonado-somatic indexes. Following measurements (weight, length, liver, and gonad), all fish as well as livers and gonads from each aquarium were pooled and frozen at −20 °C for subsequent proximate and amino acid analysis.

Prior to the analysis of the proximate composition, fish samples were dried using a VENTICELL dryer (Model LSIS-B2V/VC 404, MMM Medcenter Einrichtungen GmbH; Germany) at 70 °C for 12–24 h, until the samples were ready for grinding. The dry matter, crude protein, crude lipid, crude ash, crude fiber, gross energy and amino acids of the diets and fish samples were analysed using the MS ISO 6496:2003 [11], ISO 1871:2009 [7,10] Method 3:0 [8], ISO 5984:2002 [7,9] Method 9:0 [7], calculation (based on Method of Analysis for Nutrition Labelling, [19]; page 106 & 5) [19] and AOAC 994.12 [1] methods, respectively.

### 2.7 Calculations and statistics

The formulae used to calculate the weight gain (WG), thermal-unit growth coefficient (TGC), condition factor (CF), feed intake (FI), feed conversion ratio (FCR), hepatosomatic index (HSI), gonadosomatic index (GSI), protein efficiency ratio (PER), lipid efficiency ratio (LER), protein productive value (PPV) and energy productive value (EPV) were as follows.

WG (g) = (tank FBW(g)/final number of fish – tank IBW(g)/initial number of fish), where FBW = final average body weight (g/fish); IBW = initial body weight (g/fish).

TGC (g1/3/°C × day) = 1000 × [(FBW1/3 – IBW1/3)/Σ(T x D)], where ∑(T x D) = sum of temperature (°C) x days.

CF (g/cm^3^) = weight of fish/(length of fish)^3^ × 100.

FI (g/fish) = dry feed intake (g)/number of fish.

FCR (g/g) = FI (g)/WG (g).

HSI (%) = liver wet weight (g)/live body weight (g).

GSI (%) = gonad wet weight (g)/live body weight (g).

PER = weight gain (g)/total protein intake (g).

LER = weight gain (g)/total lipid intake (g).

PPV (g) = [final protein content of fish (g) − initial protein content of fish (g)]/total protein intake (g); EPV (kcal/100 g dm/kcal/100 g dm) = [(final energy content of fish − initial energy content of fish) (energy consumed−1)]; Data were analysed using 2-way ANCOVA in R Package (R version 3.5.0 (2018-04-23). The ANCOVA was used to correct for the difference in initial body weight between the two experimental generations of fish. The significance level was P < 0.05. When the interaction effect (fish generation × diet) was significant, the treatment means were separated using the Tukey’s HSD test. When the main effect of fish generation was significant, a Student’s t-test was applied to separate the means. When the main effect of the diet was significant, a regression analysis was applied to estimate the optimum methionine level in the diet [16,22].

## 3 Results

### 3.1 Growth, feed utilization, survival, condition factor and somatic indexes

The fish generation × diet interaction was not significant (P > 0.05) on any of the growth, feed utilization, survival, condition factor and somatic parameters studied (Table 3). This allowed the consideration of the main effects of the individual variables, which were the fish generation (also termed generation effect) and the methionine content of the diet (also termed diet effect).

**Table 3:** Growth, feed utilization, survival and somatic indexes of two generations of improved juvenile Nile tilapia juvenile tilapia fed six experimental diets containing graded levels of dietary methionine for 42 days.

	VARIABLES^a^								PARAMETERS^b^							
			IBW (g)	FBW (g)	WG (g/fish)	TGC (g1/3/°C × day)	FI (g dry matter/fish)	FCR (g dry matter/g life fish)	PER (g/g)	LER (g/g)	PPV (g/g)	EPV (kcal/100 g dm/kcal/100 g dm)	Survival (%)	CF (g/cm^3^)	HSI (%)	GSI (%)
Main effect	**Generation**^c^	G16	17.51 ^**a**^	88.78 ^**a**^	73.24 ^**a**^	1.53 ^**a**^	104.56 ^**a**^	1.43 ^**b**^	1.96	6.47 ^**b**^	1.00 ^**a**^	0.87 ^**a**^	88.33 ^**a**^	1.67	1.43	0.99 ^**b**^
		G17	19.55 ^**b**^	102.44 ^**b**^	82.93 ^**b**^	1.63 ^**b**^	112.32 ^**b**^	1.36 ^**a**^	2.13	6.90 ^**a**^	1.13 ^**b**^	0.90 ^**b**^	93.33 ^**b**^	1.67	1.06	0.67 ^**a**^
Main effect	**Diet**	0.52	18.51	96.11	77.64	1.59	105.42	1.36	2.07	6.79	1.09	0.91	93.33	1.64	1.40	0.74
		0.62	18.60	99.49	80.82	1.63	110.90	1.37	2.10	6.53	1.14	0.91	93.33	1.76	1.39	0.65
		0.84	18.53	103.36	84.87	1.68	114.13	1.35	2.17	6.53	1.10	0.87	88.33	1.71	1.11	1.46
		0.94	18.54	93.03	80.51	1.54	105.44	1.32	2.19	6.99	1.07	0.91	90.00	1.69	1.35	1.10
		1.04	18.56	91.75	73.19	1.53	114.88	1.56	1.78	6.50	0.94	0.84	88.33	1.61	1.13	0.57
		1.27	18.43	89.92	71.49	1.50	99.89	1.41	1.97	6.78	1.05	0.88	91.67	1.61	1.08	0.45
Interaction effect	**G16**	0.52	17.46	88.88	71.43	1.53	101.46	1.42	1.98	6.46	1.04	0.88	90.00	1.67	1.55	0.79
	**G16**	0.62	17.53	90.72	72.99	1.56	100.16	1.37	2.05	6.68	1.15	1.00	90.00	1.71	1.53	0.91
	**G16**	0.84	17.63	99.36	81.79	1.66	114.18	1.40	2.08	6.30	1.01	0.84	86.67	1.71	1.41	1.94
	**G16**	0.94	17.53	83.34	77.78	1.45	102.47	1.33	2.17	6.73	0.94	0.85	90.00	1.75	1.62	1.35
	**G16**	1.04	17.52	88.02	70.50	1.52	111.08	1.57	1.69	6.39	0.90	0.84	86.67	1.60	1.21	0.67
	**G16**	1.27	17.38	82.35	64.97	1.44	98.02	1.51	1.81	6.28	0.96	0.81	86.67	1.61	1.24	0.30
	**G17**	0.52	19.56	103.34	83.85	1.64	109.37	1.31	2.15	7.11	1.14	0.94	96.67	1.62	1.25	0.70
	**G17**	0.62	19.66	108.27	88.65	1.70	121.64	1.38	2.15	6.37	1.12	0.83	96.67	1.81	1.26	0.39
	**G17**	0.84	19.44	107.36	87.95	1.69	114.07	1.30	2.25	6.77	1.18	0.89	90.00	1.71	0.80	0.98
	**G17**	0.94	19.55	102.72	83.23	1.63	108.41	1.31	2.21	7.26	1.19	0.96	90.00	1.63	1.07	0.85
	**G17**	1.04	19.60	95.47	75.87	1.53	118.68	1.55	1.87	6.61	0.99	0.84	90.00	1.61	1.06	0.47
	**G17**	1.27	19.48	97.49	78.00	1.57	101.76	1.30	2.13	7.29	1.14	0.94	96.67	1.62	0.91	0.61
P-Value																
**Main effects**	**Generation**		5.46e-07 ***	5.56e-07 ***	0.019267 *	<2e-16 ***	0.0635*	0.00589 **	NS	0.00161 **	0.000501 ***	2.71e-05 ***	0.00589 **	NS	NS	1.83e-08 ***
	**Diet**		NS	0.00156 **	0.00433**	<2e-16 ***	NS	NS	0.01610 *	NS	NS	NS	NS	NS	8.44e-05 ***	NS
**Interaction effect**	Generation x Diet		NS	NS	NS	NS	NS	NS	NS	NS	NS	NS	NS	NS	NS	NS
**Pooled SEM**			3.79	2.67	2.67		0.03	0.07		0.99	0.08	0.19	0.04	0.01	0.06	0.08
Regressions																
	**Linear**															
	**Quadratic Cubic**			FBW = 259.52 (Methionine level)^3–733.97 (Methionine level)^2 + 646.49 (Methionine level) - 79.004, R^2^ = 0.8163	WG = 221.43 (Methionine level)^3^ - 647.12 (Methionine level)^2^ + 587.95 (Methionine level) - 85.4, R^2^ = 0.8792	TGC = 3.3287 (Methionine level)^3–9.4053 (Methionine level)^2 + 8.2739 (Methionine level) - 0.6539, R^2^ = 0.798			PER = 7.7806 (Methionine level)^3–21.65 (Methionine level)^2 + 18.868 (Methionine level) - 3.0341, R^2^ = 0.51						HSI = −1.4537 (Methionine level)^3 + 4.1206 (Methionine level)^2–4.1432 (Methionine level)^ + 2.6683, R^2^ = 0.6458	
	**Broken-line equation 1**			y = 21.819x + 85.252	y = 21.905x + 66.653	y = 0.283x + 1.4434			y = 0.2907x + 1.9187						y = −0.3803x + 1.5896	
				R^2^ = 0.9697	R^2^ = 0.9796	R^2^ = 0.985			R^2^ = 0.9954						R^2^ = 0.284	
	**Broken-line 2**			y = −26.331x + 121.44	y = −23.866x + 100.91	y = −0.341x + 1.9115			y = −0.5674x + 2.6054						y = −0.7214x + 1.9669	
				R^2^ = 0.6456	R^2^ = 0.7098	R^2^ = 0.6305			R^2^ = 0.2965						R^2^ = 0.7358	
**Optimum level**				0.75	0.75	0.75			0.80						1.11	

a For detailed information on the experimental diets, please refer to Table 1. G16 refers to the 16th generation of the improved GIFT Nile tilapia; G17 refers to the 17th generation of the improved GIFT Nile tilapia.

b IBW: Initial weight; FBW: final weight; WG: Weight gain; TGC: thermal-unit growth coefficient; CF: condition factor; FI: feed intake; FCR: feed conversion ratio; HSI: hepatosomatic index; GSI: gonadosomatic index; PER: protein efficiency ratio; LER: lipid efficiency ratio; PPV: Protein productive value; EPV: energy productive value. For more details on the formulae of each of these parameters, the reader is referred to the Calculation section of this paper.

c The data of the main effect of the fish generation with different superscript letters within a column are significantly different at *P < 0.05*.

The fish generation had a significant (P < 0.05) main effect on the final body weight (FBW), weight gain (WG), thermal-unit growth coefficient (TGC), feed intake (FI), feed conversion ratio (FCR), lipid efficiency ratio (LER), protein productive value (PPV), energy productive value (EPV), fish survival and gonadosomatic index (GSI) (Table 3). For each of these parameters, the 17th generation of the GIFT strain of Nile tilapia performed better than the 16th generation. This means that the application of genetic selection to produce the 17th generation effectively fish growth, feed utilization and fish survival, and delayed the development of gonad in the GIFT strain of Nile tilapia. The fish generation did not have a significant (P > 0.05) main effect on the condition factor (CF), protein efficiency ratio (PER), and hepatosomatic index (HSI) (Table 3).

The methionine content of the diet (diet effect) had a significant (P < 0.05) main effect on final body weight (FBW), weight gain (WG), thermal-unit growth coefficient (TGC), protein efficiency ratio (PER), and hepatosomatic index (HSI) (Table 3). The regression analysis revealed a significant (P < 0.05) cubic regression for each of these parameters, with the equations FBW = 259.52 (Methionine level)^3^ - 733.97 (Methionine level)^2^ + 646.49 (Methionine level) - 79.004, r^2^ = 0.8163; WG = 221.43 (Methionine level)^3^ - 647.12 (Methionine level)^2^ + 587.95 (Methionine level) - 85.4, r^2^ = 0.8792; TGC = 3.3287 (Methionine level) ^3^ - 9.4053 (Methionine level)^2^ + 8.2739 (Methionine level) - 0.6539, r^2^ = 0.798; PER = 7.7806 (Methionine level)^3^ - 21.65 (Methionine level)^2^ + 18.868 (Methionine level) - 3.0341, r^2^ = 0.51; and HSI = −1.4537 (Methionine level)^3^ + 4.1206 (Methionine level)^2^ - 4.1432 (Methionine level) + 2.6683, r^2^ = 0.6458 for the FBW, WG, TGC, PER and HSI, respectively (Table 3). When the broken analysis was applied on each of these parameters, the intersection points of the two equations, which represent the estimated optimum methionine level in the diet, showed a value of 0.75%, 0.75%, 0.75%, 0.80%, and 1.11% methionine in the diet, for the FBW, WGTGC, PER and HSI, respectively (Table 3). This data obtained with the growth parameters and PER suggest that the optimum methionine requirement level in the diet of the improved GIFT strain of Nile tilapia is between 0.75% and 0.80% of the diet. The methionine content of the diet (diet effect) did not have a significant (P > 0.05) main effect on feed intake (FI), feed conversion ratio (FCR), lipid efficiency ratio (LER), protein productive value (PPV), energy productive value (EPV), fish survival, condition factor (CF), and gonadosomatic index (GSI) (Table 3). This means that these parameters were not relevant in estimating the optimum dietary methionine requirement level in the improved GIFT strain of Nile tilapia.

### 3.2 Macronutrient and amino acid composition of the fish

Neither the fish generation × diet interaction nor the fish generation and diet had significant (P > 0.05) effects on the dry matter, crude protein, crude lipids, crude ash and gross energy contents of the whole body of the experimental fish (Table 4). This means that the macronutrient composition of the 17th and 16th generation of the improved strain of Nile tilapia used in this study remains the same, whatever the methionine content of the diet.

**Table 4:** Macronutrient composition of the whole body of two generations of improved juvenile Nile tilapia juvenile fed six experimental diets containing graded levels of dietary methionine for 42 days.

	Variables^a^	Dry Matter, dm (%)	Crude Protein (g/100 g dm)	Crude Lipid (g/100 g dm)	Crude Ash (g/100 g dm)	Crude Fiber (g/100 g dm)	Gross Energy (kcal/100 g dm)
	**Generation**	28.09	53.72	23.35	13.84	0.94	506.2
		28.28	53.85	22.91	13.91	0.97	484.53
	**Diet**	28.86	53.8	23.51	13.93	1.06	506.12
		28.02	53.68	23.32	13.67	0.96	506.78
		28.2	53.83	22.92	13.85	0.89	501.32
		28.08	54.27	23.54	14.16	1.03	454.03
		28.05	53.6	23.3	13.89	0.98	505.61
		27.9	53.54	22.2	13.77	0.82	498.33
	**G16**	29.25	53.55	23.59	13.76	1.02	508.35
	**G16**	26.98	54.01	24.03	13.74	0.95	512.48
	**G16**	28.91	53.7	23.35	13.81	1	503.69
	**G16**	28.64	53.5	23.76	14.05	0.97	506.93
	**G16**	27.72	54.08	23.55	13.88	0.96	509.1
	**G16**	27.05	53.48	21.83	13.81	0.75	496.64
	**G17**	28.47	54.05	23.43	14.09	1.1	503.89
	**G17**	29.06	53.34	22.6	13.6	0.97	501.08
	**G17**	27.49	53.97	22.49	13.9	0.79	498.95
	**G17**	27.53	55.03	23.33	14.27	1.08	401.13
	**G17**	28.38	53.11	23.06	13.9	1.01	502.12
	**G17**	28.75	53.6	22.57	13.73	0.89	500.02
**P-Value**							
**Main effects**	**Generation**	NS	NS	NS	NS	NS	NS
	**Diet**	NS	NS	NS	NS	NS	NS
**Interaction effect**	**Generation x Diet**	NS	NS	NS	NS	NS	NS
**Pooled SEM**		0.99	0.59	0.71	0.21	0.1	32.97

a For detailed information on the experimental diets, please refer to Table 1. G16 refers to the 16th generation of the improved GIFT Nile tilapia; G17 refers to the 17th generation of the improved GIFT Nile tilapia.

The fish generation × diet interaction was not significant (P > 0.05) for any of the amino acids contained in the whole body experimental fish (Table 5). This allowed the consideration of the main effects of the individual variables, the fish generation and the methionine content of the diet. The fish generation had significant (P < 0.05) main effect on the arginine, lysine, phenylalanine, alanine, glycine, glutamic acid and proline of the whole body experimental fish (Table 5). Apart from alanine, the 16th generation of the improved strain of GIFT Nile tilapia had a significantly higher content of each of these amino acids than their counterparts of the 17th generation. This suggests that the genetic improvement that led to the production of the 17th generation of GIFT tilapia reduced the contents of these amino acids in the whole body of the fish. The fish generation did not have a significant (P > 0.05) main effect on the histidine, isoleucine, leucine, methionine, threonine, valine, aspartic acid, serine and tyrosine (Table 5). This means that most of the genetic improvement affected 6 out of 16 amino acids studied.

**Table 5:** Amino acid composition of the whole body of two generations of improved juvenile Nile tilapia juvenile fed six experimental diets containing graded levels of dietary methionine for 42 days.

	Treatments^a^		Essential Amiino Acids (g/100 g dm)									Non-Essential Amino Acids						
			Arginine	Histidine	Isoleucine	Leucine	Lysine	Methionine	Phenylalanine	Threonine	Valine	Alanine	Aspartic Acid	Glycine	Glutamic Acid	Proline	Serine	Tyrosine
Main effect	**Generation**^b^	**G16**	**1.63**^**b**^	**2.33**	**0.83**	**4.42**	**4.45**	**1.55**	**3.53**^**b**^	**5.11**	**3.09**	**3.81**^**a**^	**4.22**	**4.39**^**b**^	**4.40**^**b**^	**3.51**^**b**^	**1.90**	**2.35**
		**G17**	**1.13**^**a**^	**2.23**	**0.74**	**4.01**	**3.76**	**1.74**	**2.68**^**a**^	**5.25**	**3.05**	**8.09**^**b**^	**4.87**	**4.29**^**a**^	**3.53**^**a**^	**2.45**^**a**^	**1.82**	**1.77**
Main effect	**Diet**	**0.52**	**1.96**	**2.08**	**0.71**	**3.95**	**4.81**	**1.48**	**3.21**	**3.94**	**2.78**	**3.55**	**5.20**	**4.47**	**4.63**	**2.16**	**1.85**	**1.89**
		**0.62**	**1.37**	**2.20**	**0.70**	**3.88**	**5.02**	**1.43**	**3.29**	**5.69**	**2.82**	**6.02**	**2.71**	**4.49**	**4.16**	**3.28**	**1.77**	**2.08**
		**0.84**	**1.36**	**2.52**	**0.85**	**4.44**	**3.94**	**1.48**	**2.57**	**5.84**	**3.36**	**6.88**	**3.14**	**4.58**	**3.92**	**3.84**	**1.83**	**2.76**
		**0.94**	**1.07**	**2.25**	**0.79**	**4.12**	**3.59**	**1.48**	**2.75**	**5.58**	**3.13**	**6.86**	**3.80**	**4.11**	**3.91**	**3.74**	**1.81**	**1.92**
		**1.04**	**1.15**	**2.28**	**0.79**	**4.34**	**3.82**	**1.47**	**3.61**	**4.79**	**3.11**	**6.60**	**6.03**	**4.32**	**3.62**	**2.06**	**1.88**	**1.80**
		**1.27**	**1.34**	**2.35**	**0.88**	**4.59**	**3.43**	**2.53**	**3.20**	**5.25**	**3.23**	**5.77**	**6.38**	**4.07**	**3.53**	**2.80**	**2.03**	**1.89**
Interaction effect	**G16**	**0.52**	**2.59**	**1.83**	**0.54**	**2.31**	**5.52**	**1.22**	**3.53**	**3.58**	**2.19**	**2.69**	**2.50**	**4.12**	**5.46**	**2.14**	**1.54**	**1.85**
	**G16**	**0.62**	**1.58**	**2.20**	**0.67**	**3.57**	**6.18**	**1.37**	**3.96**	**6.19**	**2.63**	**3.18**	**2.19**	**4.58**	**5.13**	**4.57**	**1.68**	**2.23**
	**G16**	**0.84**	**1.67**	**2.67**	**1.04**	**5.43**	**4.63**	**1.64**	**2.42**	**5.26**	**3.53**	**3.69**	**2.55**	**4.44**	**4.37**	**5.59**	**1.94**	**4.06**
	**G16**	**0.94**	**1.26**	**2.45**	**0.89**	**4.66**	**3.40**	**1.63**	**3.15**	**6.25**	**3.45**	**4.16**	**4.20**	**4.05**	**4.23**	**4.48**	**1.82**	**1.98**
	**G16**	**1.04**	**1.27**	**2.44**	**0.88**	**5.22**	**3.55**	**1.60**	**4.51**	**4.82**	**3.40**	**4.84**	**6.52**	**4.66**	**3.62**	**2.27**	**2.05**	**1.86**
	**G16**	**1.27**	**1.38**	**2.41**	**0.98**	**5.35**	**3.39**	**1.85**	**3.62**	**4.55**	**3.35**	**4.28**	**7.34**	**4.50**	**3.58**	**1.99**	**2.39**	**2.12**
	**G17**	**0.52**	**1.33**	**2.32**	**0.89**	**5.59**	**4.10**	**1.74**	**2.89**	**4.30**	**3.37**	**4.42**	**7.90**	**4.83**	**3.81**	**2.17**	**2.15**	**1.93**
	**G17**	**0.62**	**1.16**	**2.20**	**0.74**	**4.20**	**3.86**	**1.50**	**2.61**	**5.20**	**3.01**	**8.85**	**3.23**	**4.40**	**3.19**	**1.99**	**1.86**	**1.93**
	**G17**	**0.84**	**1.05**	**2.37**	**0.67**	**3.45**	**3.25**	**1.33**	**2.73**	**6.41**	**3.19**	**10.07**	**3.72**	**4.72**	**3.47**	**2.08**	**1.72**	**1.46**
	**G17**	**0.94**	**0.87**	**2.06**	**0.69**	**3.58**	**3.77**	**1.33**	**2.36**	**4.91**	**2.82**	**9.57**	**3.40**	**4.16**	**3.59**	**3.01**	**1.79**	**1.87**
	**G17**	**1.04**	**1.04**	**2.11**	**0.70**	**3.45**	**4.08**	**1.33**	**2.71**	**4.76**	**2.82**	**8.35**	**5.53**	**3.97**	**3.61**	**1.85**	**1.71**	**1.75**
	**G17**	**1.27**	**1.31**	**2.30**	**0.78**	**3.82**	**3.47**	**3.22**	**2.78**	**5.94**	**3.12**	**7.26**	**5.42**	**3.65**	**3.49**	**3.60**	**1.67**	**1.67**
P-Value																		
Main effects	**Generation**		0.00312 **	NS	NS	NS	0.076	NS	7.38e-05 ***	NS	NS	7.04e-10 ***	NS	7.04e-10 ***	0.000596 ***	0.0402 *	NS	NS
	**Diet**		0.02556 *	NS	NS	NS	0.00676 **	NS	NS	NS	NS	0.0213 *	0.005715 **	NS	0.005339 **	NS	NS	NS
Interaction effect	**Generation x Diet**		NS	NS	NS	NS	NS	NS	NS	NS	NS	NS	NS	NS	NS	NS	NS	NS
Pooled SEM			0.27	0.20	0.09	0.53	0.65	0.52	0.32	0.80	0.25	0.85	0.93	0.32	0.40	0.85	0.14	0.53
**Regressions**																		
	**Linear**														Glutamic acid = −1.3596x + 5.1466, R^2^ = 0.8815			
	**Quadratic**																	
	**Cubic**		Arginine = −4.6867 (Methionine level)^3 + 15.836 (Methionine level)^2–17.172 (Methionine level) + 7.1964, R^2^ = 0.8464				Lysine = 8.0267 (Methionine level)^3–19.517 (Methionine level)^2 + 12.565 (Methionine level) + 2.5565, R^2^ = 0.8961					Alanine = 39.75 (Methionine level)^3–123.06 (Methionine level)^2 + 121.99 (Methionine level) - 32.062, R^2^ = 0.9753	Aspartic acid = −91.191 (Methionine level)^3 + 260.46 (Methionine level)^2–232.66 (Methionine level) + 68.588, R^2^ = 0.9682					
	**Broken-line equation 1**		y = −1.676x + 2.6628				y = −3.0743x + 6.619					y = 9.3278× - 0.6732	y = −5.075x + 7.0328					
			R^2^ = 0.7495				R^2^ = 0.7777					R^2^ = 0.7832	R^2^ = 0.3892					
	**Broken-line equation 2**		y = 0.8337x + 0.2844				y = −0.9725x + 4.689					y = −3.3651x + 10.057	y = 7.852× - 3.1938					
			R^2^ = 0.9999				R^2^ = 0.6199					R^2^ = 0.9958	R^2^ = 0.8076					
**Optimum level**			0.95				0.92					0.85	0.79					

a For detailed information on the experimental diets, please refer to Table 1. G16 refers to the 16th generation of the improved GIFT Nile tilapia; G17 refers to the 17th generation of the improved GIFT Nile tilapia.

b The data of the main effect of the fish generation with different superscript letters within a column are significantly different at *P < 0.05*.

The methionine content of the diet (diet effect) had a significant (P < 0.05) effect on arginine, lysine, alanine, aspartic acid and glutamic acid (Table 5). The regression analysis revealed a significant (P < 0.05) cubic regression for each of these parameters, with the equations Arginine = −4.6867 (Methionine level)^3^ + 15.836 (Methionine level)^2^ - 17.172 (Methionine level) + 7.1964, r^2^ = 0.8464; Lysine = 8.0267 (Methionine level)^3^ - 19.517 (Methionine level)^2^ + 12.565 (Methionine level) + 2.5565, r^2^ = 0.8961; Alanine = 39.75 (Methionine level)^3^ - 123.06 (Methionine level)^2^ + 121.99 (Methionine level) - 32.062, r^2^ = 0.9753; and Aspartic acid = −91.191 (Methionine level)^3^ + 260.46 (Methionine level)^2^ - 232.66 (Methionine level) + 68.588, r^2^ = 0.9682 for the arginine, lysine, alanine, and aspartic acid, respectively, and a linear regression, Glutamic acid = −1.3596x + 5.1466, r^2^ = 0.8815, for the glutamic acid (Table 5).

When the broken analysis was applied on each of these parameters showing the cubic regression, the intersection points of the two equations, which represented the estimated optimum methionine level in the diet, showed a value of 0.95%, 0.92%, 0.85%, and 0.79% methionine in the diet, for the arginine, lysine, alanine and aspartic acid, respectively (Table 5). The data of the arginine, lysine, alanine and aspartic acid contents of the fish therefore suggest that the optimum methionine requirement level in the diet of the improved GIFT strain of Nile tilapia is between 0.79% and 0.95% of the diet. The linear regression observed with the glutamic acid content of the fish suggests that the more methionine there is in the diet, the less glutamic acid in the whole body of the fish. The methionine content of the diet (diet effect) did not have a significant (P > 0.05) effect on methionine, histidine, isoleucine, leucine, phenylalanine, threonine, valine, tyrosine, proline, glycine and serine (Table 5). This means that the content of these amino acids in the whole body of fish is not relevant in estimating the optimum dietary methionine requirement level in the improved GIFT strain of Nile tilapia.

## 4 Discussion

The present study aimed at investigating whether or not the genetic improvement affected the dietary methionine requirement level to support the gain in growth in Nile tilapia. When growth parameters (FBW, WG and TGC) were considered, the 17th generation performed 15% better than the 16th generation, which suggested that the genetic improvement program implemented on the 16th generation to produce the 17th generation of the GIFT strain of Nile tilapia was effective [3,15]. This result was consistent with the 10–15% increase in fish weight previously recorded from one generation to the next one with the GIFT genetic improvement program [15]. The higher growth overserved in the 17th generation was supported by the better FI, FCR, LER, PPV and EPV observed in this group of fish, compared with the 16th generation. These results suggest that while selecting for fast growth, the genetic improvement program that was applied to the GIFT strain of Nile tilapia used in this experiment has likely also selected the fish for higher feed efficiency [5]. However, further studies, including a more standardized way to assess feed intake are warranted to confirm this assumption [17].

Moreover, there was no significant interaction between the fish generation and the dietary methionine levels studied in this experiment. Thus, whatever the generation of GIFT tilapia considered, the results show that the optimum dietary methionine requirement in terms of growth of the improved GIFT strain of Nile tilapia was estimated between 0.75% and 0.80% of the diet (with a cysteine level of 0.50% of the diet), which is aligned with 0.80% of the diet (with 0.2% cysteine in the diet) observed in tilapia of 0.06 g initial body weight by Ref. [18]; prior to the start of the GIFT genetic improvement program in 1988 [3,15]. However, the fact that the cysteine level at the optimum methionine requirement level in this experiment was 2.5 times higher than the cysteine level reported by Ref. [18] suggests that the genetic improvement program led to a higher requirement of the total sulfur amino acids, although the methionine requirement level did not specifically changed.

In contrast, when the growth parameters were again considered, the dietary methionine requirement level observed in this study was 60% higher than that observed in juvenile Nile tilapia of 1.3 g initial body weight by Ref. [13]. This increase in the dietary methionine requirement level, at the same cysteine level in the diet as in Ref. [13]; likely confirms the assumption that the improved GIFT strain of the Nile tilapia might have a methionine or total sulfur amino acids requirement higher than the conventional strain. These observations are in line with the review published by Ref. [12]; which highlighted significant differences in nutrient utilization and requirements between the improved (GIFT in this case) and non-improved strains of tilapia. These authors added that it was warranted to update the existing databases with information on the higher nutrient requirements of improved strains of tilapia, in order to ensure that diets are accurately formulated to allow the full expression of their accrued growth potential, which was gained through genetic improvement. Furthermore, there is currently a general trend to update the nutrient requirements of improved strains of animals in general, considering that their genetic improvement has made them more demanding of nutrients and use them more efficiently to increase their growth rate [4,6].

The lower HSI observed in the 17th generation than the 16th generation of the GIFT strain of Nile tilapia, whereas the GSI was similar between these two groups of fish, suggesting that the fish of the 16th generation used in this experiment were likely approaching the maturation stage, as HSI usually increases with the onset of reproduction [2]. In other words, the genetic selection might have delayed the maturation in the 17th generation of the GIFT strain of Nile tilapia, to the benefit of growth. This assumption is supported by the fact that in the present experiment the methionine requirement level for the highest HSI is estimated at 1.11% of the diet, which is higher than that leading to the highest growth of fish (0.75–0.8% of the diet).

Although there was no significant main or interactive effect of fish generation and diets on the macronutrients (dry matter, crude protein, crude lipid and crude ash) and gross energy contents of the fish in this study, the fact that the fish of the 17th generation showed a significantly lower lysine, phenylalanine, arginine, proline, glycine and glutamic acid contents in their whole body experimental fish than the 16th generation of the improved strain of GIFT Nile tilapia is difficult to explain. Nevertheless, the optimum methionine requirement levels in the diet of the improved GIFT strain of Nile tilapia that were estimated between 0.79% and 0.95% of the diet for the lysine, arginine, aspartic acid and alanine contents of the fish are well aligned with the estimates obtained with growth data.

## 5 Conclusion

∗ The genetic improvement led 15% more growth in the 17th generation than the 16th generation of the GIFT strain of Nile tilapia, which was accompanied with higher FI, FCR, LER, PPV and EPV in the former group;
∗ Whatever the generation considered, the optimum dietary methionine requirement in terms of growth of the improved GIFT strain of Nile tilapia of 17–19 g IBW was estimated between 0.75% and 0.80% of the diet (with a cysteine level of 0.50% of the diet), which is higher than the 0.49% of the diet previously estimated for conventional Nile tilapia at the juvenile stage.;
∗ The genetic improvement applied to the GIFT strain of Nile tilapia led to a higher methionine (and total sulfur amino acids) requirement level in this strain than what is reported for the conventional strains;

The results of this study support the need to update the existing nutrient requirement databases of fish, in order to ensure that diets are formulated to effectively satisfy the requirements of not only conventional, but also improved strains of fish, for the full expression of their accrued growth potential of the latter strains.

## Author contribution statement

Rodrigue Yossa: Conceived and designed the experiments; Performed the experiments; Analysed and interpreted the data; Contributed reagents, materials, analysis tools or data; Wrote the paper.

Nurulhuda Ahmad Fatan; Kirthigah Palanivelu: Performed the experiments; Analysed and interpreted the data; Contributed reagents, materials, analysis tools or data; Wrote the paper.

## Funding statement

Rodrigue Yossa was supported by CGIAR Research Program on Fish Agri-Food Systems (FISH) [CGIAR-FISH-CRP].

## Data availability statement

Data will be made available on request.

## Declaration of interest's statement

The authors declare no conflict of interest.
