# Corrigendum to “Revaluating the dietary methionine requirements in an improved strain of Nile tilapia (Oreochromis niloticus)” [Heliyon 9 (3), (March 2023) Article e14376]

**DOI:** 10.1016/j.heliyon.2023.e17614

**Published:** 2023-06-29

**Authors:** Rodrigue Yossa, Nurulhuda Ahmad Fatan, Kirthigah Palanivelu

**Affiliations:** Sustainable Aquaculture, WorldFish, Jalan Batu Maung, Batu Maung, 11960 Bayan Lepas, Penang, Malaysia

In the original published version of this article, the wrong reference list was published. To fix this, a corrected reference list will be published. The correct version of the reference list can be found below. The publisher apologize for the errors. Both the HTML and PDF versions of the article have been updated to correct the errors.

The correct reference list:
